# Hsa-miR-34a mediated repression of corticotrophin releasing hormone receptor 1 regulates pro-opiomelanocortin expression in patients with complex regional pain syndrome

**DOI:** 10.1186/s12967-016-0820-1

**Published:** 2016-03-03

**Authors:** Botros B. Shenoda, Guillermo M. Alexander, Seena K. Ajit

**Affiliations:** Pharmacology and Physiology, Drexel University College of Medicine, 245 North 15th Street, Mail Stop 488, Philadelphia, PA 19102 USA; Neurology, Drexel University College of Medicine, Philadelphia, USA

**Keywords:** MicroRNA, Complex regional pain syndrome, Ketamine, Pain, hsa-miR-34a, Pro-opiomelanocortin, Corticotropin-releasing hormone

## Abstract

**Background:**

Ketamine provides relief for a subset of patients with complex regional pain syndrome (CRPS). The poor responders had a lower body mass index (BMI) relative to responders. Regulation of proopiomelanocortin (POMC) expression is crucial in normal body weight homeostasis. The main objectives of this study were to investigate the mechanisms underlying lower BMI characterizing CRPS patients responding poorly to intravenous ketamine therapy and identify potential biomarkers for predicting response.

**Methods:**

We investigated POMC transcript levels in blood from CRPS patients grouped as responders and poor responders to ketamine therapy. Plasma levels of β-endorphin, ACTH and α-MSH were measured by ELISA. We previously identified differential expression of small noncoding microRNA hsa-miR-34a in blood between responders and poor responders. We investigated whether a 11-fold downregulation of hsa-miR-34a in poor responders relative to responders is contributing to the differences in POMC levels by targeting POMC regulator CRHR1. Binding of miR-34a to CRHR1 was assessed using reporter assay; changes in mRNA and protein levels of CRHR1 were used to determine the regulation of CRHR1 by miR-34a. RNA from blood of CRPS and control subjects were used for quantitative PCR for CRHR1.

**Results:**

Though ketamine treatment did not alter POMC expression, poor responders had higher levels of POMC mRNA than responders, both before and after treatment. Corticotropin-releasing hormone (CRH) is a key regulator of POMC expression and the biological effects are mediated through its receptor corticotrophin releasing hormone receptor 1 (CRHR1). We show that hsa-miR-34a is a negative regulator of CRHR1; overexpression of hsa-miR-34a in Jurkat cells resulted in reduction of CRH-mediated POMC expression. Poor responders had higher expression of CRHR1 transcripts than responders, indicating a regulatory role for miR-34a. In addition, we found positive correlations between the pretreatment levels of miR-34a to BMI and response to ketamine therapy.

**Conclusions:**

Our findings indicate a mechanism by which hsa-miR-34a can regulate the CRH/CRHR1/POMC axis and may influence BMI. Studies in larger patient cohorts are required to confirm the biomarker utility of circulating hsa-miR-34a levels in predicting treatment response to ketamine therapy.

**Electronic supplementary material:**

The online version of this article (doi:10.1186/s12967-016-0820-1) contains supplementary material, which is available to authorized users.

## Background

Complex regional pain syndrome (CRPS) is a chronic neuropathic pain condition characterized by a broad array of symptoms including inflammation, trophic disturbances, and sensory and motor dysfunction [1–3]. CRPS is one of the most difficult chronic pain disorders to treat partly due to incomplete understanding of its pathophysiologic mechanisms. Ketamine is used to treat CRPS patients refractory to standard therapy [4]. However, approximately a third of patients fail to respond to ketamine treatment [5]. Our data have shown that poor responders to ketamine therapy had lower body mass index (BMI) relative to responders [6].

Pro-opiomelanocortin (POMC) is a precursor polypeptide with 241 amino acid residues that is cleaved posttranslationally in a tissue-specific manner to a number of bioactive peptide hormones, including adrenocorticotrophic hormone (ACTH), α-melanocyte-stimulating hormone (α-MSH), and β-endorphin [7, 8]. The POMC gene is predominantly expressed in the anterior and intermediate lobes of the pituitary and its mRNA has been detected in several other tissues, including the brain, lymphocytes, skin, testis, thyroid, placenta, pancreas, gut, kidney, adrenal, and liver [9]. POMC-expressing cells centrally and peripherally play a major role in mediating pain and energy homeostasis [10]. β-Endorphin, derived from POMC, when secreted from immune cells peripherally at sites of inflammation occupies the opioid receptors on sensory nerves and causes analgesia by inhibiting neuronal excitability [11]. POMC-related melanocortin peptides are essential for regulating body weight, appetite, and energy expenditure, and reduction in POMC expression is associated with increased body weight [12, 13]. Here, we examined POMC mRNA levels in blood samples from patients with CRPS including responders and poor responders to ketamine and observed that POMC expression was significantly higher in poor responders.

MicroRNAs (miRNAs) are small noncoding RNAs that can negatively regulate gene expression by binding to the 3′ untranslated region (3′UTR) of mRNAs, inducing mRNA degradation or translational repression [14]. Recently, we investigated the differential expression of miRNAs in the blood of responders as compared to poor responders prior to and after ketamine therapy. Hsa-miR-34a showed a 11-fold downregulation in poor responders relative to responders prior to treatment, indicating underlying molecular differences that may determine treatment response [6].

Corticotropin-releasing hormone (CRH) acts as a key regulator of POMC gene expression. CRH enhances POMC transcription in vivo and in vitro [15]. The biological effects of CRH are mediated by at least two different types of G-protein–coupled receptor, CRHR1 and CRHR2. CRH exhibits 10 times higher affinity toward CRHR1 than toward CRHR2 [16], and CRHR1 is essential for mediating the effects of CRH on POMC expression [17]. Bioinformatics analysis showed that CRHR1 mRNA is a predicted target for miRNA-34a [18]. We hypothesized that the reduced expression of miR-34a could contribute to the increased expression of POMC observed in poor responders to ketamine therapy by regulating CRHR1.

## Methods

### Inclusion and exclusion criteria

All subjects were enrolled after giving informed consent as approved by the Drexel University College of Medicine Institutional Review Board. All patients met the clinical Budapest criteria for CRPS [19]. A total of 19 control subjects and 27 CRPS patients included in this study; 15 CRPS patients received intravenous ketamine (Additional file 5: Table S1). Eligibility for ketamine treatment, treatment protocol, and response assessment were discussed in detail before [6]. Patients were grouped as responders if following treatment their average pain score decreased by at least 50 % and poor responders if their average pain score either increased or decreased less than 50 %.

### RNA isolation and qPCR for POMC and CRHR1 in blood

Total RNA was isolated from whole blood as described previously [6]. cDNA was synthesized using 500 ng total RNA using Maxima First Strand cDNA Synthesis Kit (Thermo Fisher Scientific, Waltham, MA). Relative expressions of POMC and of CRHR1 were determined using qPCR. The Assay IDs for the Taqman primer probes were POMC (Hs01596743_m1) and CRHR1 (Hs00366363_m1), and 18S was used as the normalizer (Applied Biosystems, Foster City, CA).

### Plasma levels of β-endorphin, ACTH, and α-MSH

ELISA was used to determine the plasma levels of ACTH (ALPCO Diagnostics, Salem, NH), β-endorphin (MD Bioproducts, St Paul, MN) and α-MSH (Phoenix Pharmaceuticals, Burlingame, CA) according to the manufacturers’ instructions. The numbers of samples from patients and controls used in these assays depended on the availability of plasma samples.

### Cell culture

HEK293 cells (American Type Culture Collection, Manassas, VA) were maintained in complete media [1× Dulbecco’s Modified Eagle Medium, 10 % heat-inactivated fetal bovine serum (FBS), and 1 % penicillin–streptomycin]. Jurkat cells were maintained in RPMI 1640 medium with 10 % FBS and 1 % penicillin–streptomycin.

### Luciferase reporter assay

The 3′UTR clones for human CRHR1 (HmiT060383) and the precursor miRNA expression clone miR-34a (HmiR0005) were purchased from GeneCopoeia (Rockville, MD). Firefly and Renilla luciferase activity were measured 24 h after transfection of HEK293 cells according to the manufacturer’s protocol.

### Transcriptional regulation of CRHR1 by miR-34a

Jurkat cells were transfected with miR-34a using the nucleofection protocol (Lonza, Walkersville, MD). RNA was isolated 24 h after transfection using a mirVana RNA isolation kit (Ambion, Austin, TX), and cDNA was generated using the Maxima First Strand cDNA Synthesis Kit. The relative expression of CRHR1 was determined using qPCR with 18S as the normalizer.

### Western blot

To determine CRHR1 protein expression 100 µg protein lysates were used. Primary antibodies used were rabbit anti-CRHR1 (1: 200, Sigma-Aldrich, St. Louis, MO) and mouse anti-GAPDH (1: 1000, Santa Cruz Biotechnology, Dallas, TX). Secondary antibodies used were goat anti-rabbit Alexa 680 and goat anti-mouse Alexa 680 (1:10,000, LI-COR Biosciences, Lincoln, NE). Blots were scanned using an Odyssey Infrared Imaging System (LI-COR Biosciences).

### CRH stimulation of Jurkat cells

Jurkat cells transfected with miR-34a were incubated with 100 nM of CRH (Sigma-Aldrich) for 2 h. RNA isolation and cDNA synthesis were performed as described above. POMC expression was determined using qPCR in miR-34a and control miRNA transfected Jurkat cells before and after CRH stimulation. 18S was used as the normalizer.

### Statistical analysis

One-way analysis of variance (ANOVA), Student t test, and Spearman and Pearson Correlation were used to perform statistical analysis. Data are expressed as mean ± SEM. A value of p < 0.05 was considered significant. GraphPad Prism software was used for all statistical analysis.

## Results

### Differences in BMI between ketamine responders and poor responders and potential predictive validity

**Fig. 1:**
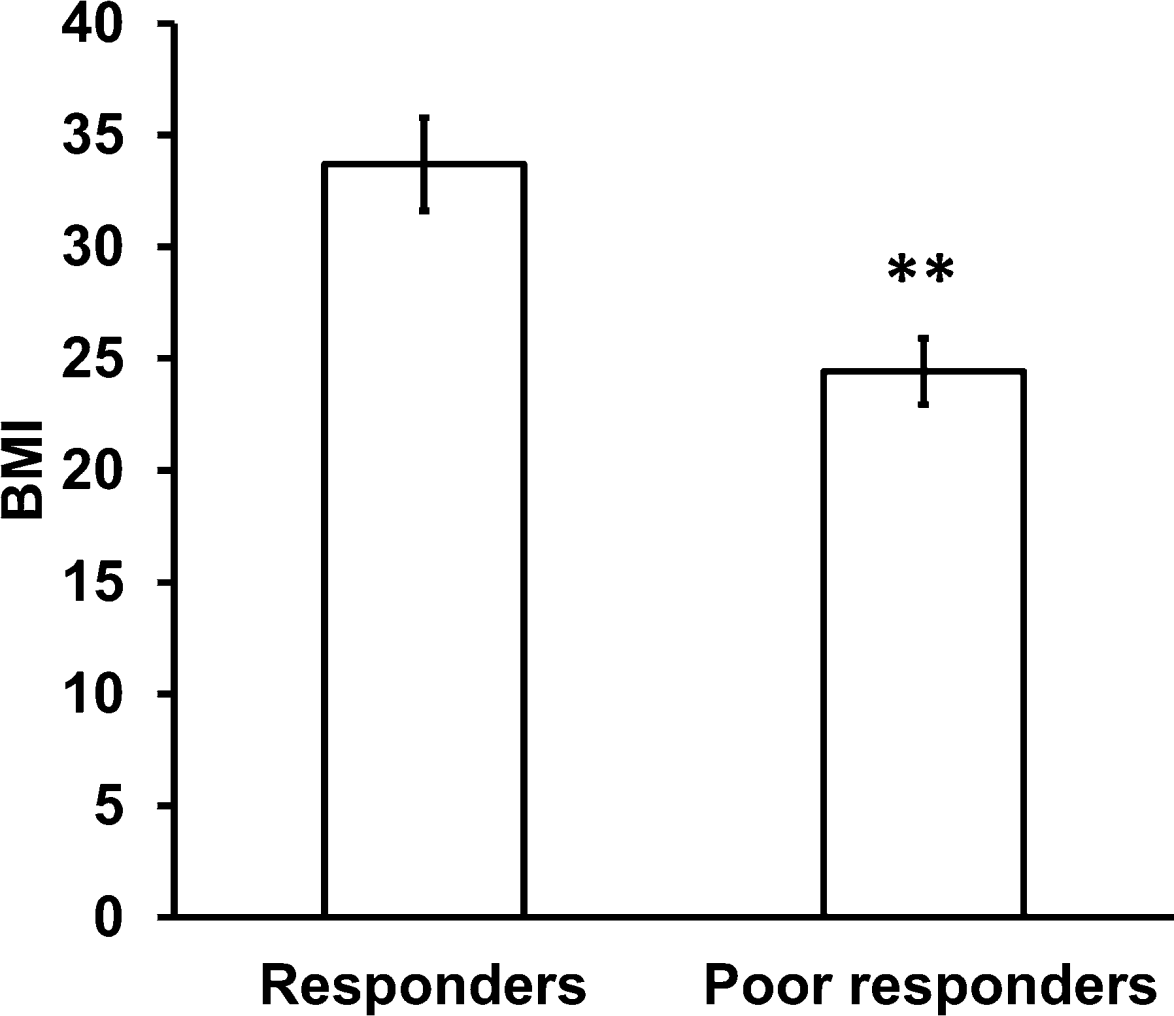
BMI of CRPS patients receiving ketamine therapy **p < 0.01 (responders, n = 8; poor responders, n = 7). Statistical analysis was conducted using unpaired t-test. Data represent mean ± SEM. See Additional file 1: Figure S1 for frequency distribution

We calculated BMI for responders and poor responders to ketamine therapy, using the formula body weight in kilograms/height in square meters. Poor responders had significantly lower body weight and BMI than responders (Fig. 1, Additional file 5: Table S1 and Additional file 1: Figure S1). Ketamine induced pain relief was positively correlated with BMI (Spearman r = 0.69, p < 0.01) and body weight (Spearman r = 0.766, p < 0.001). Although the main goal of this study was to understand the molecular mechanisms that may contribute to the observed difference in BMI, we were interested in assessing the role of body weight in predicting the analgesic response of CRPS patients to ketamine therapy. Thus, we analyzed the body weight and BMI in two independent cohorts of CRPS patients from previous studies [20, 21]. In the first study [21], the treatment regimen was identical to ours (Additional file 5: Table S1 and Additional file 2: Figure S2) whereas the second study by Schwartzman et al., 2009 differed in that the patients received a lower dose of ketamine in an outpatient setting for 10 days (Additional file 5: Table S1 and Additional file 3: Figure S3). Combining data from all patients including this study show that poor responders to ketamine therapy have significantly lower body weight relative to responders (Additional file 5: Table S1 and Additional file 4: Figure S4).

### Expression of POMC in the blood of patients with CRPS

**Fig. 2:**
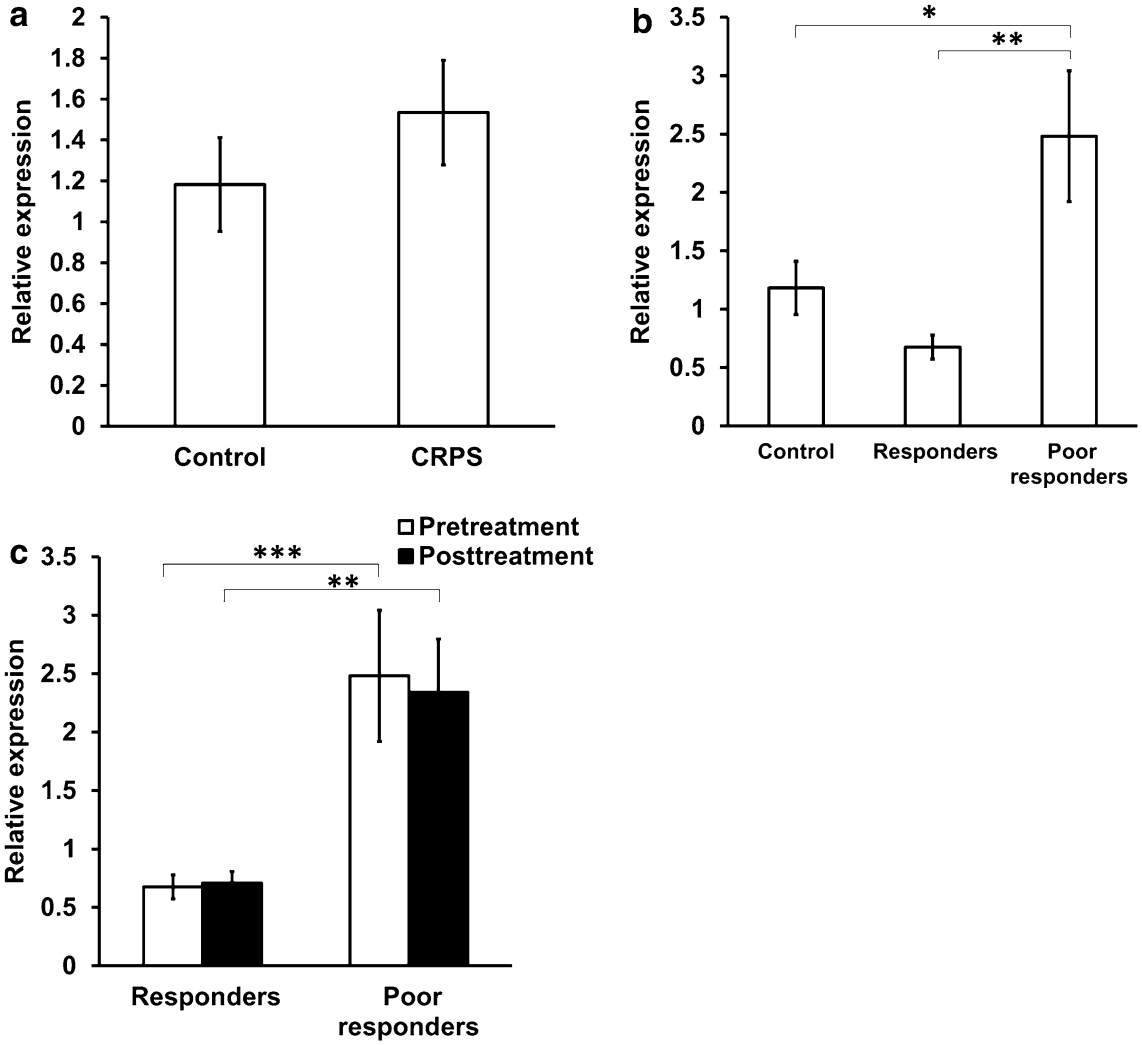
Expression of POMC in CRPS patients. **a** Relative expression of POMC mRNA in the blood of CRPS patients (n = 26) and healthy controls (n = 19). 18S was used as the normalizer gene. Statistical analysis was conducted using unpaired t-test. **b** Relative expression of POMC in the blood of CRPS patients prior to ketamine therapy (responders, n = 8; poor responders, n = 7) and healthy controls (n = 19). 18S was used as the normalizer.*p < 0.05 **p < 0.01. Statistical analysis was conducted using one-way ANOVA with Bonferroni multiple comparison test as post-Hoc test. **c** Changes in relative expression of POMC in the blood of CRPS patients in response to ketamine therapy (responders, n = 8; poor responders, n = 7). Data were normalized to 18S expression. **p < 0.01, ***p < 0.001. Statistical analysis was conducted using paired and unpaired t-test. For all *panels*, data represent mean ± SEM

POMC mRNA levels were determined in whole blood from patients with CRPS. Although reports have shown dysregulated endogenous opioid peptides in CRPS [22, 23] we did not observe statistically significant differences in the expression of POMC between patients with CRPS and a control population (Fig. 2a). However, when we categorized the CRPS population according to response to ketamine therapy, we found that poor responders to ketamine therapy showed higher expression of POMC than responders and control subjects prior to treatment (Fig. 2b). Ketamine induced pain relief was negatively correlated with pretreatment POMC expression levels (Spearman r = −0.68, p < 0.01). Ketamine therapy did not significantly alter the expression of POMC in either responders or poor responders (Fig. 2c).

### Plasma β-endorphin, ACTH, and α-MSH levels in patients with CRPS

**Fig. 3:**
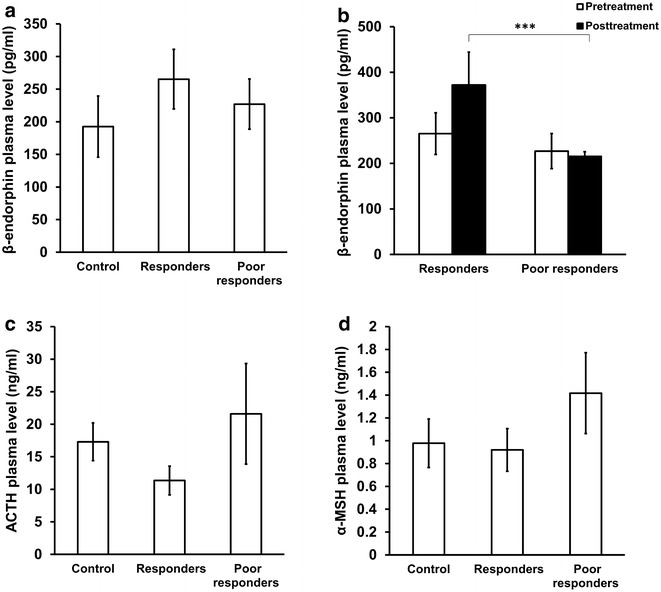
Plasma levels of β-endorphin, ACTH, and α-MSH in CRPS patients **a** Plasma levels of β-endorphin in CRPS patients prior to ketamine therapy (responders, n = 8; poor responders, n = 7; healthy controls, n = 6). Statistical analysis was conducted using one-way ANOVA with Bonferroni multiple comparison test as post-Hoc test. **b** Changes in β-endorphin levels in the plasma of CRPS patients in response to ketamine therapy (responders, n = 8; poor responders, n = 7). *** p < 0.001. Statistical analysis was conducted using paired and unpaired t-test. **c** Plasma levels of ACTH in patients prior to ketamine therapy (responders, n = 8; poor responders, n = 7; healthy controls, n = 6). Statistical analysis was conducted using one-way ANOVA with Bonferroni multiple comparison test as post-Hoc test.** d** Plasma levels of α-MSH in CRPS patients prior to ketamine therapy (responders, n = 8; poor responders, n = 7; healthy controls, n = 6). Statistical analysis was conducted using one-way ANOVA with Bonferroni multiple comparison test as post-Hoc test. For all panels, data represent mean ± SEM

We investigated the plasma levels of POMC derived peptides β-endorphin, ACTH, and α-MSH in patients to determine whether the differential expression of POMC mRNA observed in ketamine responders and poor responders is reflected on the plasma level of POMC-derived peptides. Our results show that β-endorphin levels did not differ between responders and poor responders before treatment. Posttreatment levels of β-endorphin were significantly higher in responders than in poor responders (Fig. 3a, b). Pretreatment ACTH values did not show significant differences among the groups (Fig. 3c). Pretreatment α-MSH levels were higher in poor responders than in responders, but the difference was not statistically significant (Fig. 3d).

### CRHR1-mediated POMC expression is regulated by miR-34a

**Fig. 4:**
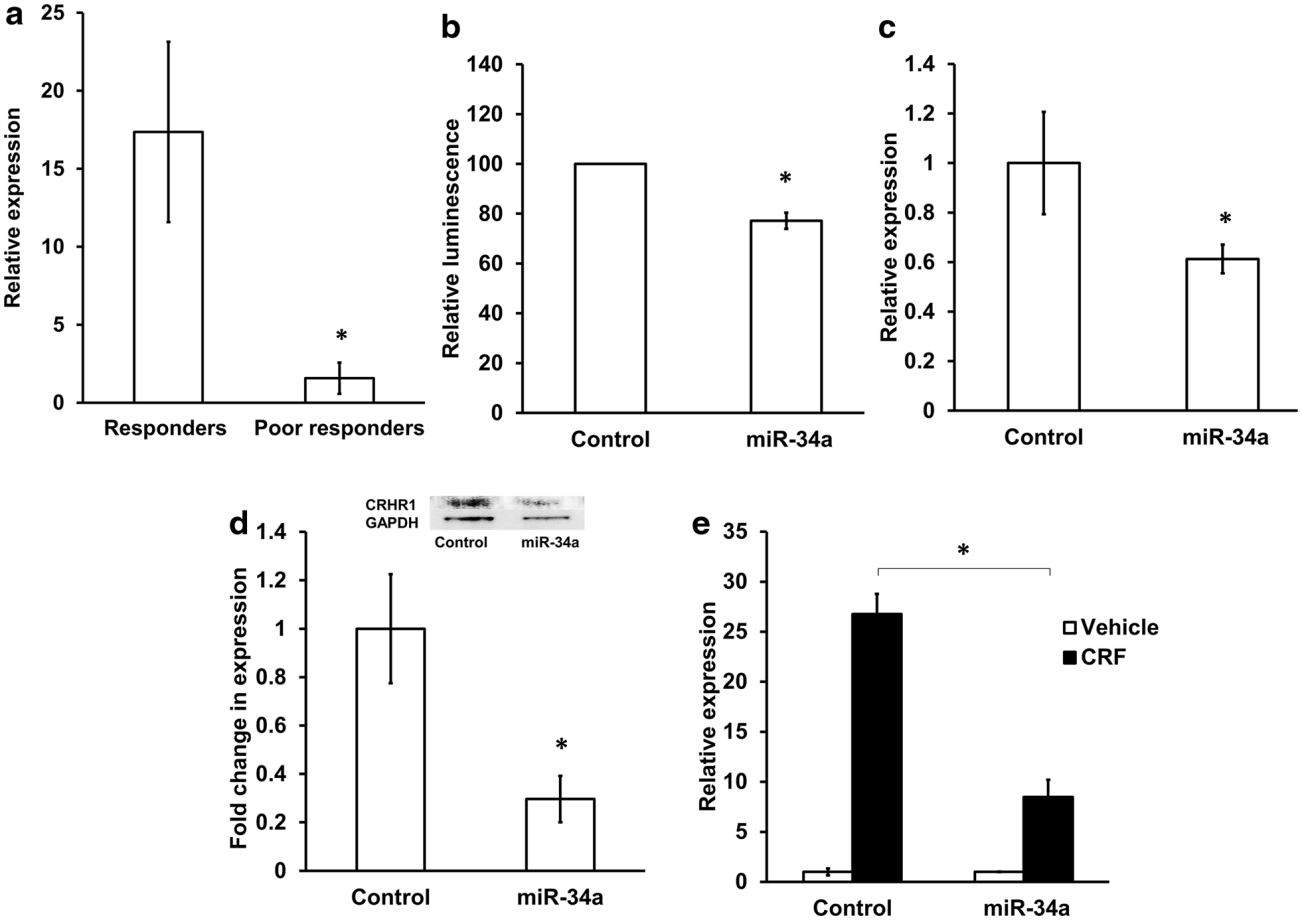
Regulation of CRHR1 by miR-34a. **a** Relative expression of miR-34a in the blood of CRPS patients (Responders n = 8, Poor responders n = 7) **b** Reporter assay confirming binding of miR-34a to the 3′UTR of CRHR1. Luciferase reporter assay was performed 24 h after cotransfection of HEK293 cells with CRHR1 3′UTR reporter plasmid and miR-34 or control miRNA. **c** Overexpression of miR-34a in Jurkat cells decreased endogenous CRHR1 mRNA. Jurkat cells were transfected with miR-34a or control miRNA. The relative expression of CRHR1 was determined using qPCR 24 h after transfection and 18S was used as the normalizer. **d** miR-34a decreased CRHR1 protein expression in Jurkat cells. Western blot analysis of CRHR1 expression in Jurkat cells 48 h after miR-34a or control miRNA transfection. Protein expression was normalized to GAPDH (n = 4, representative blot shown). **e** miR-34a can repress the CRH-induced increase in POMC expression. Jurkat cells transfected with miR-34a or control miRNA were incubated with 100 nM of CRH for 2 h. POMC expression was determined using qPCR before and after CRH stimulation. 18S was used as the normalizer. All in vitro data represent an average of three or more independent experiments. *p < 0.05. Unpaired t-test was used for statistical analysis. For all *panels*, data represent mean ± SEM

miRNAs negatively regulate gene expression by binding to the 3′UTR of target mRNAs to induce translational repression or mRNA degradation [14]. Bioinformatics prediction showed that CRHR1 is a potential target of miRNA-34a, a miRNA that is downregulated 11 fold in poor responders relative to responders prior to treatment (Fig. 4a) [6]. miR-34a has a role in lipid metabolism and obesity [24, 25]. These factors along with the positive correlation between miR-34a and ketamine-induced pain relief (Spearman r = 0.56, p < 0.05) (Additional file 5: Table S1) lead us to select miR-34a for further investigation. We hypothesized that the reduced expression of miR-34a in poor responders could contribute to an increase in POMC expression by regulating CRHR1. A luciferase reporter assay confirmed the binding of miR-34a to the 3′UTR of CRHR1 (Fig. 4b). Jurkat cells (human lymphocyte cell line) transfected with miR-34a showed reduced expression of endogenous CRHR1 mRNA and protein compared with cells transfected with control miRNA (Fig. 4c, d). Moreover, miR-34a reduced the CRH-induced increase in POMC expression (Fig. 4e). These in vitro studies show that a decrease in miR-34a might contribute to the increased expression of POMC mRNA in poor responders.

### Expression of CRHR1 in the blood of patients with CRPS

**Fig. 5:**
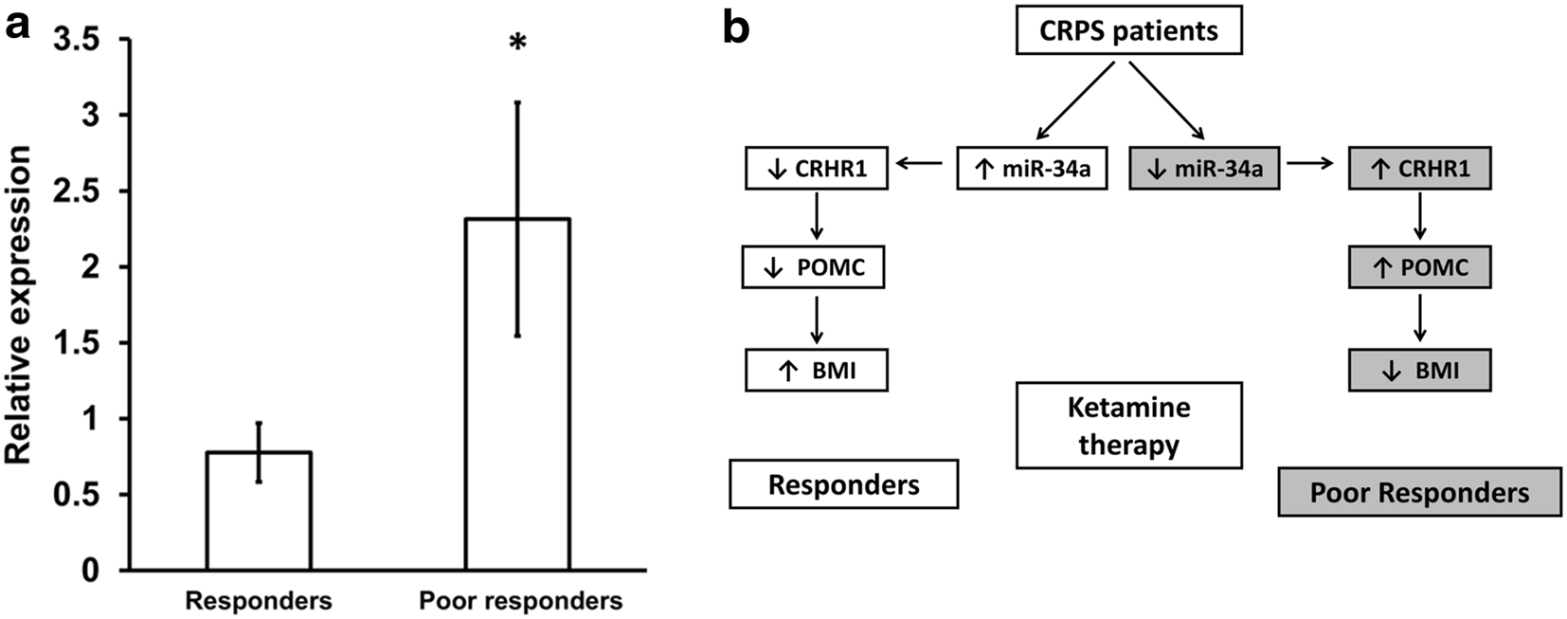
CRHR1 alterations in CRPS patients and schematic representation of miR-34a mediated repression of CRHR1 regulating POMC expression and BMI **a** CRHR1 was significantly higher in poor responders than in responders. Relative expression of CRHR1 mRNA in blood from responders (n = 8) and poor responders (n = 7) to ketamine treatment. Data were normalized to 18S. *p < 0.05. Unpaired t-test was used for statistical analysis. Data represent mean ± SEM. **b** Grouping of CRPS patients based on miR-34a expression, BMI and response to ketamine. miR-34a may influence BMI by regulating CRHR1/POMC pathway

Because POMC expression is mediated by CRHR1 activity, and our in vitro data showed that miR-34a can regulate CRHR1, we investigated the expression of CRHR1 in the blood of responders and poor responders to ketamine to confirm the regulatory role of miR-3a on CRHR1. CRHR1 mRNA was significantly higher in poor responders compared to responders (Fig. 5a), confirming an inverse correlation in expression between miR-34a and its mRNA target CRHR1.

## Discussion

Ketamine has a well-established role in the treatment of acute and chronic pain disorders, including neuropathic conditions [4]. Many studies have shown that sub-anesthetic dose of ketamine could be beneficial in treating refractory CRPS [26, 27]. It has been shown that ketamine elicited different molecular responses in CRPS patients suggesting that the commonality in disease symptoms doesn’t necessarily indicate dysregulation of same molecular pathways [6]. We observed that the poor responders to ketamine had lower BMI than responders. In addition to having lower BMI, the poor responders to ketamine had increased POMC expression. It is likely that altered expression of POMC may mediate the observed difference in BMI. POMC and its related melanotropic peptides, especially α-MSH, play important roles in regulating body weight [28]. This is mediated centrally within the hypothalamus [29, 30] by increasing energy expenditure and weight loss. The contribution of peripherally expressed POMC to obesity, however, is still largely unexplored. The expression of POMC has been demonstrated in blood cells including lymphocytes at both the mRNA and protein level [31]. The functional significance of POMC expression in lymphocytes is still not entirely clear, and researchers generally assume that it forms part of a biochemical loop linking the immune, nervous, and endocrine systems [31, 32]. Processing of POMC in lymphocytes is similar to that observed in the anterior pituitary [31, 32], and hence we relied on a human lymphocyte cell line i.e., Jurkat cells for our in vitro experiments. Investigating centrally dysregulated POMC and related pathways in obese subjects may require extremely invasive maneuvers and sampling. Thus studying POMC signaling in more accessible systems such as peripheral blood cells might be reflective of central events and can provide insight into signaling mechanisms underlying obesity.

Previous work has suggested that α-MSH is the most important POMC-derived peptide responsible for POMC-mediated weight reduction. Produced in the arcuate nucleus of the hypothalamus, α-MSH activates melanocortin-4 receptor promoting satiation and energy expenditure [29, 30] reducing body weight [33]. Although the role of central α-MSH in regulating body weight is well understood, the correlation of circulating α-MSH with body weight is less clear. The source of circulating α-MSH in humans remain unclear, as it is produced by different organs, including inflammatory cells [34]. Plasma α-MSH was not significantly different between obese and non-obese normal or hypertensive individuals [35]. In another study, plasma and cerebrospinal fluid α-MSH levels also did not correlate significantly with weight, BMI, or body composition [36]. In other studies, however, α-MSH correlated positively with BMI [37]; thus findings are not consistent across reports.

Elucidating the mechanisms inducing differential expression of POMC in CRPS patients can be beneficial in understanding ketamine-induced analgesia in these patients. Ketamine potentiates opioid analgesia, prevents opioid-induced acute tolerance, interacts with opioid receptors, and increases the release of β-endorphin [38]. Though sub-anesthetic dose of ketamine produces its analgesic effect through modulation of the endogenous opioid system, it is known that higher doses of ketamine can antagonize the effects of morphine [39]. It is not known whether ketamine can modulate the expression of POMC and thereby the levels of its downstream peptide β-endorphin, an important mediator of the endogenous analgesic system [40]. In our studies ketamine did not affect transcriptional regulation of POMC because the mRNA levels were not altered after therapy. Posttreatment levels of β-endorphins were significantly higher in responders than in poor responders. This suggests that ketamine-induced modulation of the endogenous antinociceptive effect may not occur at the transcriptional level of POMC but through increasing β-endorphin release thus contributing to the opioid-potentiating effect of ketamine in CRPS [41].

The mechanism(s) underlying the higher expression of POMC in poor responders in not known. Although POMC expression can be regulated through a negative feedback mechanism mediated by endogenous opioids and mu receptor agonists [42–45] this seem to be unlikely in CRPS patients as the basal (pre-treatment) levels of POMC was higher in poor responders relative to responders despite comparable basal levels of β-endorphin. CRH acts as a key regulator of POMC gene expression by stimulating its transcription [15, 46]. The biological effects of CRH are mediated by two different types of GPCR, CRHR1 and CRHR2. CRH exhibits 10 times higher affinity toward CRHR1 than toward CRHR2, and CRHR1 is essential for mediating the effects of CRH on POMC expression [17]. Poor responders show downregulation of miR-34a [6], which is predicted to target and reduce the expression of CRHR1. So we hypothesized that reduced miR-34a in poor responders may increase CRHR1 and subsequent upregulation of POMC. Our results confirmed this hypothesis in showing that miR-34a can bind to the 3′UTR of CRHR1 and reduce its mRNA and protein levels. The ability of CRH to induce the expression of POMC was significantly impaired in Jurkat cells overexpressing miR-34a. Poor responders to ketamine therapy also had higher CRHR1 and thus may contribute to higher levels of POMC in this group. Taken together, these data indicate that miR-34a can target and reduce the expression of CRHR1, thereby impairing the CRH/CRHR1/POMC axis and may contribute to altered regulation of body weight.

In addition to its role in regulating POMC expression, CRHR1 is involved in inflammatory responses. CRHR1 is upregulated in macrophages upon LPS stimulation; blocking of CRHR1 prolonged survival in mice with endotoxic shock and significantly reduced endotoxin-induced proinflammatory cytokines [47]. We hypothesize that the pharmacological blockade using CRHR1 antagonists may be beneficial in pain management for a subset of CRPS patients. Though we do not have evidence on the role of CRHR1 on ketamine analgesia in particular, it is worth exploring whether a combinatorial approach can augment the analgesic efficacy of ketamine or other therapeutics in treatment resistant patients. Chronic blockade of CRHR1 with systemic Antalarmin, a CRHR1 receptor specific antagonist, significantly ameliorated incomplete Freund’s adjuvant induced inflammation, and weight loss associated with disease onset in susceptible LEW/N rats [48], suggesting a role for CRHR1 mediated signaling in regulating body weight in inflammatory states. Based on our finding that CRHR1 is upregulated in poor responders, further investigations on the role of CRHR1 signaling in this particular group of CRPS patients is warranted including alterations in inflammatory mediators.

## Conclusions

Collectively, our in vitro studies demonstrated that miR-34a, which was downregulated in poor responders, can regulate CRHR1 and CRH-mediated POMC expression. Expression of CRHR1, an important inducer of POMC expression, was also upregulated in poor responders. Our data support the hypothesis that reduced expression of miR-34a could contribute to the increased expression of POMC observed in poor responders to ketamine therapy and regulate the CRH/CRHR1/POMC axis (Fig. 5b). Lower levels of miR-34a could be one factor underlying increased POMC expression and reduced body weight suggesting a potential utility of BMI, POMC, CRHR1 and miR-34a as surrogate markers, predictors or biomarkers of ketamine response. Body weight can be used in initial assessment for predicting ketamine response but our finding requires confirmation in larger patient cohorts. Further studies are needed to confirm the proposed role of miR-34a in regulating the CRH/CRHR1/POMC axis in pain-free obese and lean subjects. The presence of stable circulating miRNAs in body fluids and the noninvasive nature of sample procurement has greatly enhanced interest in pursuing miRNAs as novel biomarkers [49]. Unlike cancer biology, where researchers have access to tissue samples, central nervous system disorders including pain are dependent on biological markers that can be identified from bodily fluids, predominantly blood. Our studies on miR-34a highlights how alterations in circulating miRNAs can provide insights on underlying disease biology.

## Additional files

10.1186/s12967-016-0820-1 Frequency distribution of BMI of poor responders and responders (A) to ketamine therapy. Body weight of CRPS patients receiving ketamine therapy grouped according to response (B). Statistical analysis was conducted using unpaired t-test. Data represent mean ± SEM **p < 0.01.
10.1186/s12967-016-0820-1 Body weight (A) and BMI (B) of CRPS patients receiving 5 days of ketamine infusion (40 mg/h) grouped according to response (n = 6 responders and ten poor responders [21]. Statistical analysis was conducted using unpaired t-test. Data represent mean ± SEM.
10.1186/s12967-016-0820-1 Body weight of CRPS patients receiving sub-anesthetic doses ketamine for 4 h daily for 10 days (maximum ketamine infusion rate was 0.35 mg/kg/h, 25 mg/h over a 4 h period) in an outpatient setting grouped according to response (n = 3 responders and 6 poor responders) [20]. Statistical analysis was conducted using unpaired t-test. Data represent mean ± SEM.
10.1186/s12967-016-0820-1 Body weight of CRPS patients stratified according to ketamine response pooled from three independent studies including this study, Goldberg et al. 2010, and Schwartzman et al. 2009 (n = 17 responders and 23 poor responders) ***p < 0.001. Statistical analysis was conducted using unpaired t-test. Data represent mean ± SEM.
10.1186/s12967-016-0820-1 Patient demographics and correlation studies. Sheets 1 and 2. Control and patient information including clinical parameters; Sheet 3. Key; Sheet 4. Pain relief determined using McGill and NRS scores of CRPS patients and their average percent change after receiving ketamine therapy; Sheet 5. Correlation analysis for relative expression of miR-34a, POMC mRNA levels, pain relief, BMI and body weight; Sheets 6 and 7. We analyzed the body weight and BMI reported for two independent cohorts of CRPS patients from previous studies [20, 21]. Sheet 6. Patient information from Goldberg et al. 2010. The treatment regimen in this study was identical to ours; Sheet 7. Patient information from Schwartzman et al. 2009. Treatment regimen differed from our study in that the patients received a lower dose (25 mg/h daily) of ketamine in an outpatient setting for 10 days. In our study ketamine was infused to patients with refractory CRPS over 5.5 days as described [6]. The infusion rate began at 10 mg/h and was increased by 10 mg/h every 2 h until a maximum rate of 40 mg/h was reached. This rate was continued for 120 h and was then tapered off by 10 mg/h every 2 h; Sheet 8. Combining data from all patients including this study show that poor responders to ketamine therapy have significantly lower body weight relative to responders.

## Abbreviations

ACTH: adrenocorticotrophic hormone
BMI: body mass index
CRH: corticotrophin releasing hormone
CRHR1: corticotrophin releasing hormone receptor 1
CRPS: complex regional pain syndrome
miRNA: microRNA
α-MSH: α-melanocyte-stimulating hormone
POMC: pro-opiomelanocortin
3′UTR: 3′ untranslated region
