# Supplementary figures and images for: Hsa-miR-34a mediated repression of corticotrophin releasing hormone receptor 1 regulates pro-opiomelanocortin expression in patients with complex regional pain syndrome
Source: J Transl Med. 2016 Mar 3;14:64. doi: 10.1186/s12967-016-0820-1 (PMC4778288; doi:10.1186/s12967-016-0820-1)

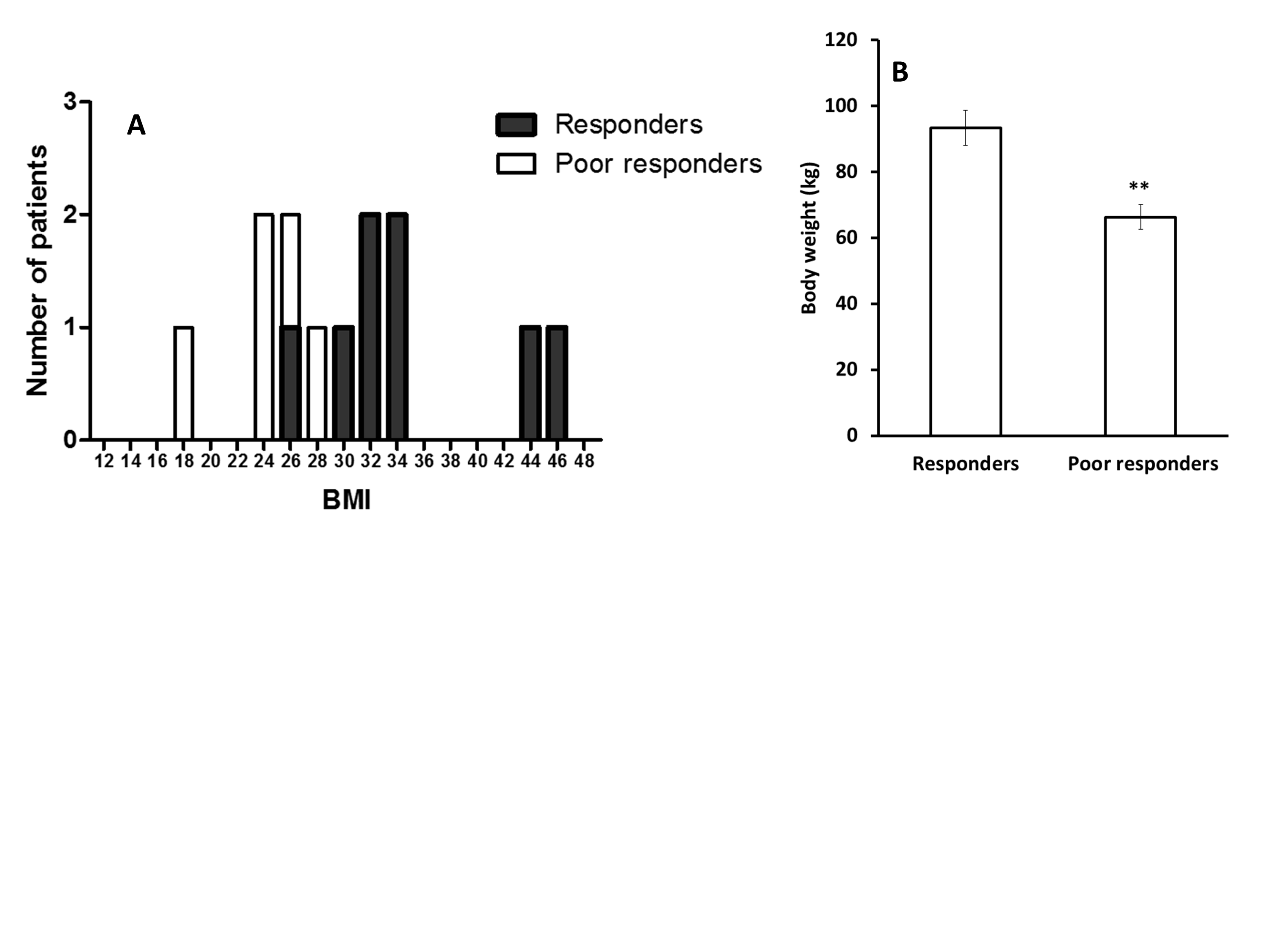

Supplement: Supplementary file 1 — 10.1186/s12967-016-0820-1 Frequency distribution of BMI of poor responders and responders (A) to ketamine therapy. Body weight of CRPS patients receiving ketamine therapy grouped according to response (B). Statistical analysis was conducted using unpaired t-test. Data represent mean ± SEM **p < 0.01. [file 12967_2016_820_MOESM1_ESM.tiff]

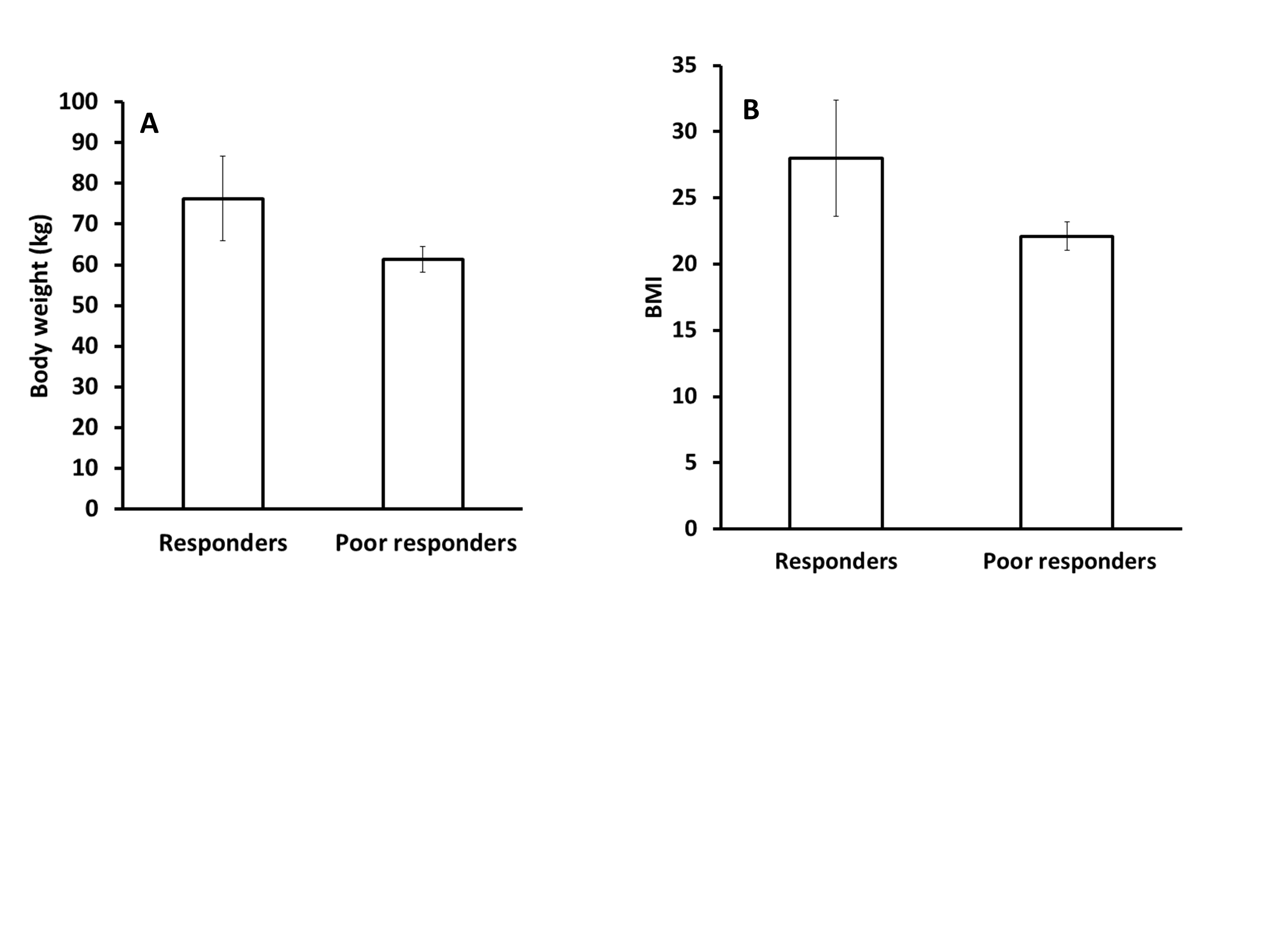

Supplement: Supplementary file 2 — 10.1186/s12967-016-0820-1 Body weight (A) and BMI (B) of CRPS patients receiving 5 days of ketamine infusion (40 mg/h) grouped according to response (n = 6 responders and ten poor responders [21]. Statistical analysis was conducted using unpaired t-test. Data represent mean ± SEM. [file 12967_2016_820_MOESM2_ESM.tiff]

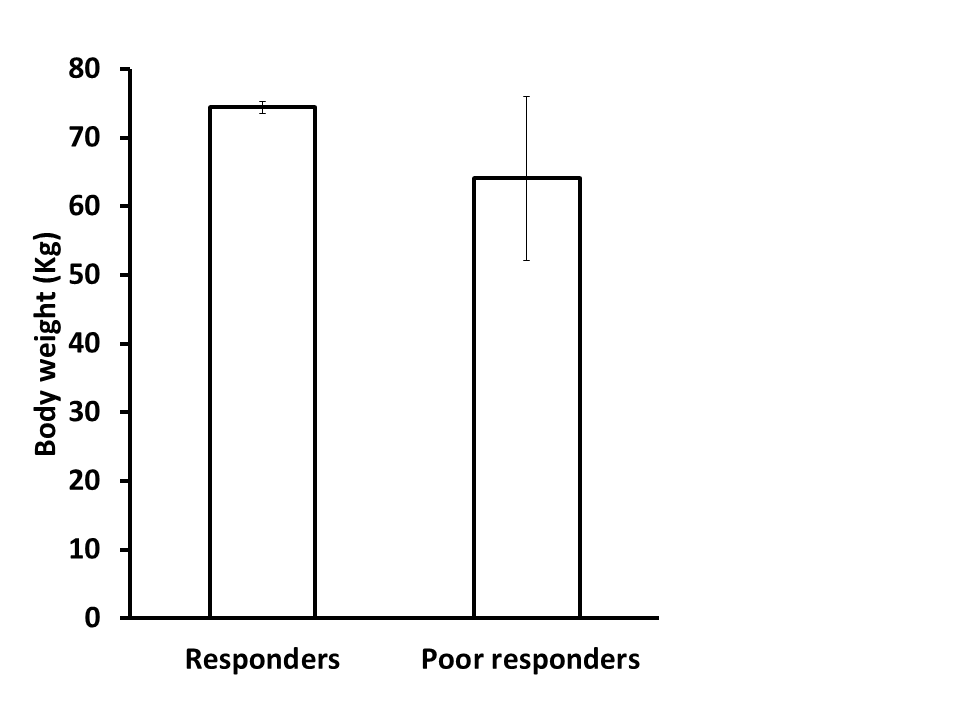

Supplement: Supplementary file 3 — 10.1186/s12967-016-0820-1 Body weight of CRPS patients receiving sub-anesthetic doses ketamine for 4 h daily for 10 days (maximum ketamine infusion rate was 0.35 mg/kg/h, 25 mg/h over a 4 h period) in an outpatient setting grouped according to response (n = 3 responders and 6 poor responders) [20]. Statistical analysis was conducted using unpaired t-test. Data represent mean ± SEM. [file 12967_2016_820_MOESM3_ESM.tiff]

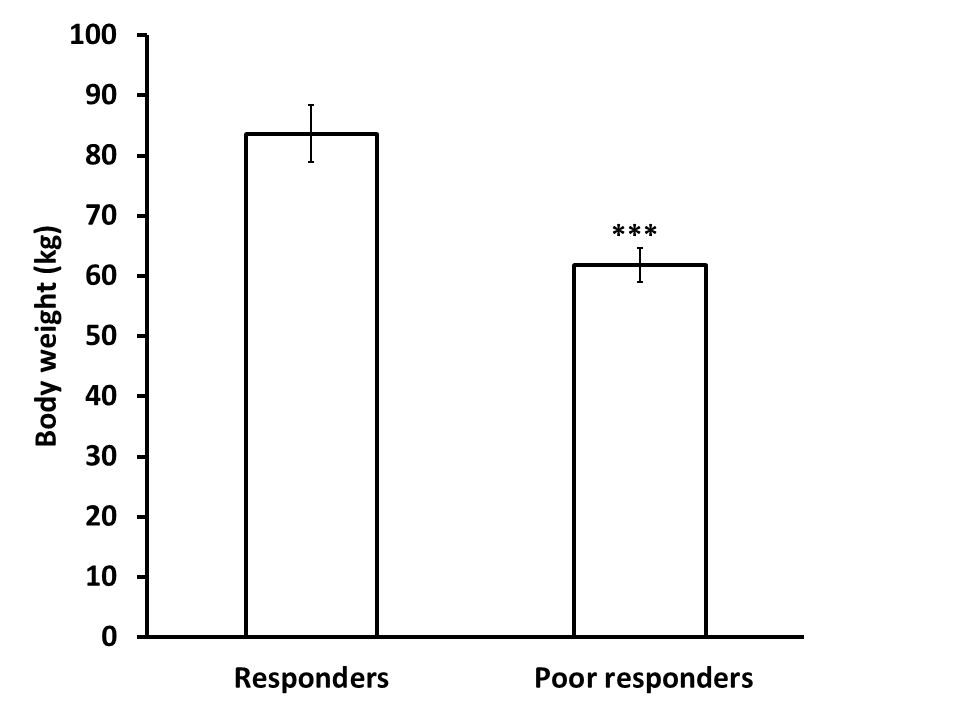

Supplement: Supplementary file 4 — 10.1186/s12967-016-0820-1 Body weight of CRPS patients stratified according to ketamine response pooled from three independent studies including this study, Goldberg et al. 2010, and Schwartzman et al. 2009 (n = 17 responders and 23 poor responders) ***p < 0.001. Statistical analysis was conducted using unpaired t-test. Data represent mean ± SEM. [file 12967_2016_820_MOESM4_ESM.tiff]
